# Synergistic Effect of Ginsenoside Rh2 Combines with Ionizing Radiation on CT26/*luc* Colon Carcinoma Cells and Tumor-Bearing Animal Model

**DOI:** 10.3390/ph16091188

**Published:** 2023-08-22

**Authors:** Shan-Chih Lee, Chao-Yu Shen, Wei-Hsun Wang, Yen-Po Lee, Keng-Wei Liang, Ying-Hsiang Chou, Yeu-Sheng Tyan, Jeng-Jong Hwang

**Affiliations:** 1 Department of Medical Imaging and Radiological Sciences, Chung Shan Medical University, Taichung 40201, Taiwan; sclee@csmu.edu.tw (S.-C.L.); hideka.chou@gmail.com (Y.-H.C.); 2 Department of Medical Imaging, Chung Shan Medical University Hospital, School of Medicine, Chung Shan Medical University, Taichung 40201, Taiwan; shenchaoyu@gmail.com (C.-Y.S.); james095797@gmail.com (K.-W.L.); 3 Department of Orthopedic Surgery, Changhua Christian Hospital, Changhua 50044, Taiwan; cmch10011@gmail.com; 4 Department of Biomedical Imaging and Radiological Sciences, National Yang Ming Chiao Tung University, Taipei Branch, Hsinchu City 30010, Taiwan; louis0952571827@gmail.com; 5 Department of Radiation Oncology, Chung Shan Medical University Hospital, Taichung 40201, Taiwan

**Keywords:** colorectal cancer, ginsenoside Rh2, radiotherapy, immunomodulation

## Abstract

Background: The local tumor control rate of colon cancer by radiotherapy is unsatisfactory due to recurrence and radioresistance. Ginsenoside Rh2 (Rh2), a panoxadiol saponin, possesses various antitumor effects. Methods: CT26/*luc* murine colon carcinoma cells and a CT26/*luc* tumor-bearing animal model were used to investigate the therapeutic efficacy of Rh2 combined with ionizing radiation and the underlying mechanisms. Results: Rh2 caused cell cycle arrest at the G1 phase in CT26/*luc* cells; however, when combined with ionizing radiation, the cells were arrested at the G2/M phase. Rh2 was found to suppress the activity of NF-κB induced by radiation by inhibiting the MAPK pathway, consequently affecting the expression of effector proteins. In an in vivo study, the combination treatment significantly increased tumor growth delay time and overall survival. Furthermore, the combination treatment significantly reduced NF-κB and NF-κB-related effector proteins, along with PD-1 receptor expression. Additionally, Rh2 administration led to increased levels of interleukin-12, -18, and interferon-γ in the mice’s sera. Importantly, biochemical analysis revealed no toxicities associated with Rh2 alone or combined with radiation. Conclusions: The combination of Rh2 with radiation may have potential as an alternative to improve the therapeutic efficacy of colorectal cancer.

## 1. Introduction

Colorectal cancer (CRC) ranks as the third leading cause of mortality in Taiwan [1]. Neoadjuvant chemoradiation therapy (nCRT) is a widely adopted treatment for CRC [2,3]. However, about 80–90% of patients with CRC did not achieve a complete response to nCRT because of the development of radioresistance in the tumor. Some cell signaling pathways are involved in radiation-induced DNA damage repair. Ionizing radiation can activate mitogen-activated protein kinase (MAPK) via Ras-Raf signaling and the growth factor receptor-bound protein 2 (Grb2) signaling and son of sevenless (SOS) signaling pathways [4]. MAPK inhibition has been shown to reduce DNA damage repair in DU145 prostate cancer cells [5]. Ataxia-telangiectasia mutated gene protein (ATM) senses damaged DNA and activates the IκB kinase to release the cytosolic NF-κB from IκB, and subsequently translocated into the nucleus through the canonical pathway [6]. NF-κB and its downstream effector proteins are critical for inducing radioresistance in several types of cancer. NF-κB promotes tumor angiogenesis and metastasis by elevating the expressions of vascular endothelial growth factor (VEGF) and matrix metalloproteinase (MMP), increasing cyclin D1 expression for tumor cell proliferation and Bcl-2 for anti-apoptosis and epithelial/mesenchymal transition (EMT) and survival [7].

Inflammation has been recognized as a risk factor for cancer formation, especially via activating the nuclear factors NF-κB and STAT3 and inducing COX-2 production [8,9]. The tumor microenvironment contains tumor-infiltrated cells, such as CD4+, CD8+ T-cells, and regulatory T-cells (Treg), which are crucial for establishing the host immune system against the tumor. Both interleukin-12 (IL-12) and -18 activate NK cells, which may suppress tumor metastasis [10]. IL-12 could induce T helper type I (Th1) immune responses, as well as upregulate classes I and II major histocompatibility (MHC) molecules and induce the expression of interferon gamma (IFN-γ) [11,12]. Tumor growth inhibition, pulmonary metastatic reduction, and increase of IFN-γ in the circulation have been reported in tumor-bearing mice after IL-12 injection [13]. IL-18 can also upregulate the expression of Fas ligand (FASL) in NK and T cells and facilitate the killing effect of target cells [14]. In addition, cRel, a NF-κB family member, plays an important role for IFN-γ transcription via induction of IL-12 and IL-18 [15].

In Taiwan, some cancer patients not only receive conventional surgery, radiotherapy, or chemotherapy but also take herbal medicine, especially ginseng, as an adjuvant [16]. The interaction and underlying mechanisms of combining current clinical treatments with herbal medicine are worth elucidating. Ginseng has been used as a medicine for more than 2000 years. Many studies have shown the pharmacological effects of ginsenoside Rh2 (Rh2), an important component of ginseng [17]. Rh2 has demonstrated anti-tumor activity by inducing G1 cell cycle arrest, promoting differentiation, and facilitating apoptosis and autophagy through the downregulation of microRNA-638 in various cancer cell types [18,19,20]. Inhibition of cell migration, invasion, angiogenesis, and epithelial-mesenchymal transition (EMT) by Rh2 have also been reported [21,22]. Moreover, Rh2 has been shown to improve immunological response by increasing lymphocyte infiltration in tumors, as observed in a melanoma mice model [23,24].

The programmed cell death protein-1 (PD-1) and cytotoxic T-lymphocyte-associated protein 4 (CTLA-4) are named “immune checkpoints” due to their role in downregulating T-cell activation. Tumor cells may escape immune surveillance using these two inhibitors through several mechanisms [25]. Clinical studies have revealed that blocking CTLA-4 and PD-1 can enhance therapeutic efficacy against several types of cancers [26,27]. Rh2 mainly displays its anti-tumor activity by inducing apoptosis and directly killing tumor cells [28,29]. Furthermore, it may suppress tumor cells by enhancing the immune response [30]. Therefore, the roles that PD-1 and CTLA-4 play in the regulation of immune function by Rh2 may also be the key factors against tumor progression. Here, we used murine colon carcinoma CT26/*luc* cells and a CT26/*luc* tumor-bearing animal model to investigate the radiosensitization of Rh2 when combined with ionizing radiation. We also examined the underlying mechanisms involved. The results revealed a synergistic effect of combining Rh2 with radiation, which holds potential benefits for the treatment of colorectal cancer in clinical settings.

## 2. Results

### 2.1. Cytotoxicity and Radiosensitization of Rh2 in CT26/luc Cells

**Figure 1:**
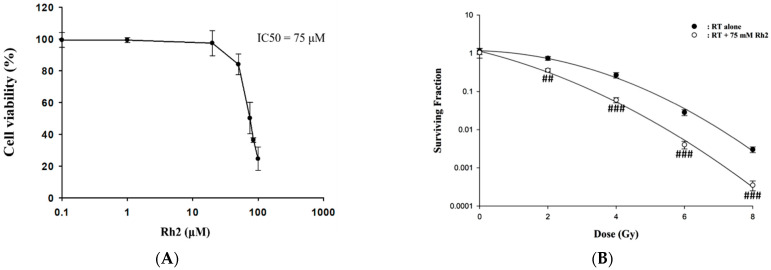
The cytotoxicity of Rh2 in CT26/*luc* cells and survival of CT26/*luc* cells treated with radiation alone or radiation combined with Rh2. (**A**) 1 × 10^4^ CT26/*luc* cells per well were seeded into a 96-well plate for 24 h. The cells were then treated with various concentrations of Rh2 for 24 h. The cell viability of CT26/*luc* cells was evaluated with an alamarBlue assay. The cytotoxicity of Rh2 on CT26/*luc* cells was in a dose-dependent manner, and the IC_50_ was 75 µM. (**B**) Rh2 enhanced radiation-induced cytotoxicity in CT26/*luc* cells in synergism. CT26/*luc* cells were pretreated with or without 75 µM Rh2 for 24 h and then treated with various radiation doses (0, 2, 4, 6, 8 Gy). The surviving fraction was assayed with colony formation. Rh2 significantly enhanced radiation-induced cell killing compared with the radiation-alone group. ^##^ *p* < 0.01; ^###^ *p* < 0.001 for the surviving fraction with Rh2 compared with that without Rh2 under the same radiation dose.

Cells were treated with 0–100 µM Rh2 for 24 h, and cell viability was compared with that of the control (0 µM), as shown in Figure 1A. The results showed that Rh2 decreased the cell viability in a dose-dependent manner with an IC_50_ of 75 µM, which was used in this study. CT26/*luc* cells were irradiated with various doses (0–8 Gy) and then incubated with or without 75 µM Rh2 for 24 h. The surviving fractions were obtained by colony formation assay. Figure 1B shows that the surviving fractions of the combination group were significantly lower than those of the radiation groups. The combination effect was calculated as presented in Table 1, and a synergistic effect was found with 75 µM Rh2 plus radiation for doses between 2 and 8 Gy.

### 2.2. Rh2 Induces G1 Arrest and Enhances G2/M Arrest When Combined with Radiation in CT26/luc Cells

**Figure 2:**
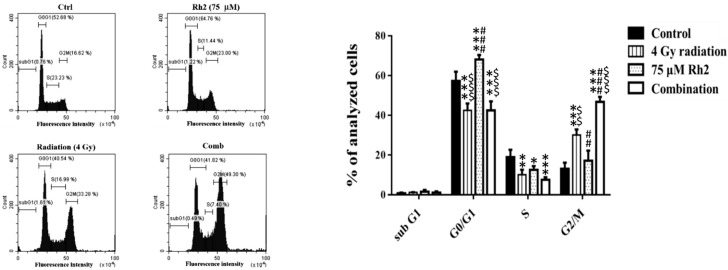
Cell cycle analysis of CT26/luc cells treated with 4 Gy radiation and 75 µM Rh2 combined with 4 Gy. CT26/luc cells (1 × 10^6^) were plated on 10-cm dishes for 24 h and then treated with 75 μM Rh2, 4 Gy radiation (at 36 h post inoculation), and a combination of both. The cell cycle was significantly arrested at the G1 phase by 75 µM Rh2. The cell population in the G2/M phase increased most significantly in the combination group. * *p* < 0.05, ** *p* < 0.01, and *** *p* < 0.001 for the experimental groups compared with that of the control. ^##^ *p* < 0.01 and ^###^ *p* < 0.001 for Rh2 alone and the combination group compared with that of the radiation-alone group. ^$$^ *p* < 0.01 and ^$$$^ *p* < 0.001 for the radiation-alone and combination groups compared with that of the Rh2-alone group.

The cell cycle of CT26/*luc* cells treated with Rh2 and radiation was analyzed by flow cytometry. Rh2 significantly arrested the cell cycle at the G1 phase (Figure 2). CT26/*luc* cells treated with the combination of 75 µM Rh2 and 4 Gy radiation resulted in S-phase reduction and significantly increased the G2/M population.

### 2.3. Cytotoxicity of Rh2 Combines with Radiation

**Figure 3:**
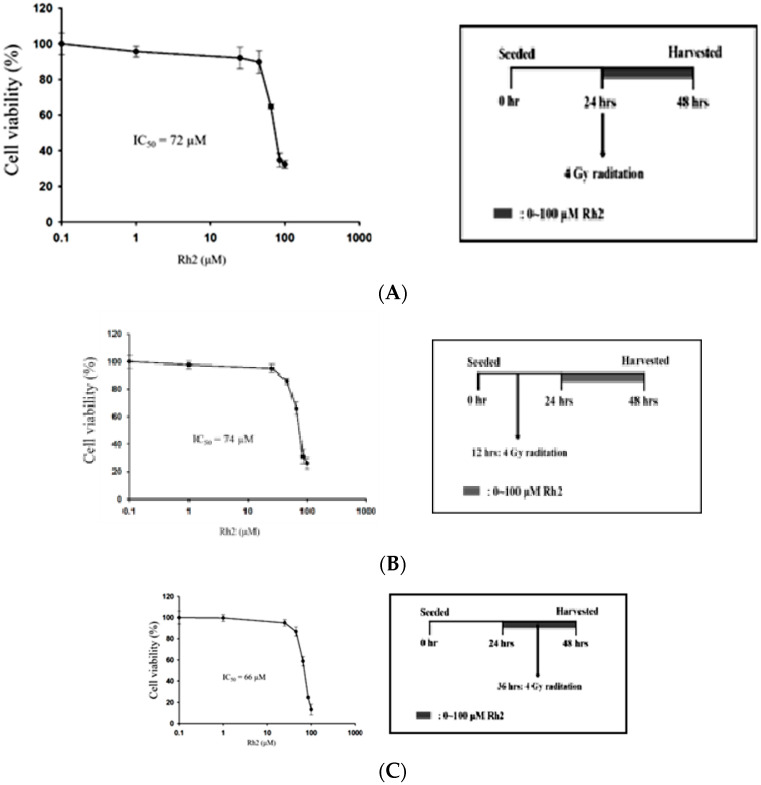
Combination effects of Rh2 with radiation. (**A**) CT26/*luc* cells were concurrently treated with Rh2 and 4 Gy radiation. (**B**) CT26/*luc* cells were irradiated with 4 Gy at 12 h before being treated with Rh2. (**C**) CT26/*luc* cells were treated with Rh2 for 12 h, followed by 4 Gy radiation. Cell viability was evaluated using the alarmarBlue assay. The results demonstrated that CT26/*luc* cells pretreated with Rh2 for 12 h, followed by 4 Gy radiation, had the lowest IC_50_ of 66 μM. The duration of Rh2 treatments was 24 h for all three combinations.

The efficacy of concurrent, post-treatment, and pretreatment in CT26/*luc* cells treated with Rh2 plus radiation was determined using the alamarBlue assay (Figure 3). The IC_50_ was 72 µM, 74 μM, and 66 μM for cells treated with Rh2 and 4 Gy radiation concurrently (Figure 3A), Rh2 after 4 Gy radiation (Figure 3B), and pretreatment with Rh2, followed by 4 Gy radiation (Figure 3C), respectively.

### 2.4. NF-κB Activity Induced by Radiation Is Suppressed by Rh2 in CT26/luc Cells

**Figure 4:**
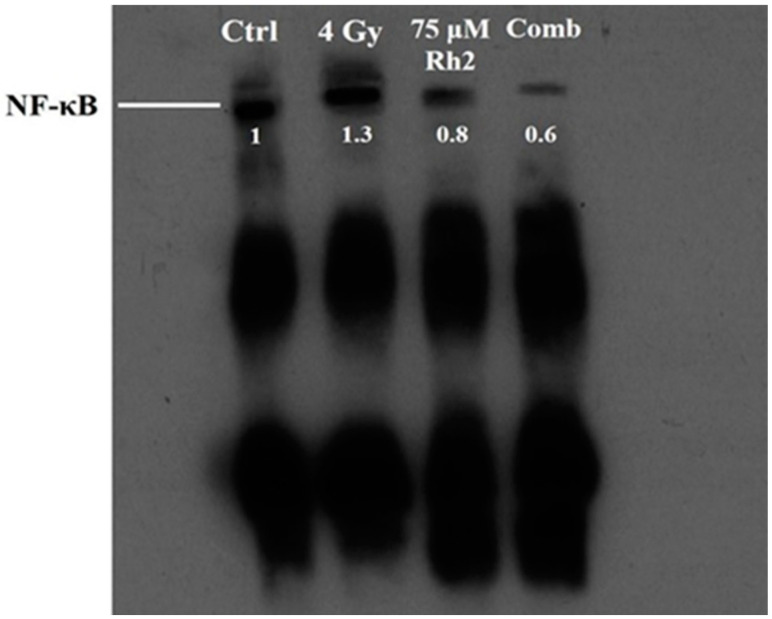
Rh2 suppresses radiation-induced NF-κB activity in CT26/*luc* cells. CT26/*luc* cells (2 × 10^6^) were seeded into the 10-cm dish for 24 h. Cells were then treated with 75 μM Rh2, 4 Gy, or 75 μM Rh2 combined with 4 Gy. Nuclear extracts were prepared, and the NF-κB activity was evaluated with EMSA. NF-κB was activated by 4 Gy radiation but suppressed by Rh2 and a combination in CT26/*luc* cells. The NF-κB levels in CT26/*luc* cells were 1.3-, 0.8-, and 0.6-fold for radiation alone, Rh2 alone, and combination, respectively, as compared with that of the control.

Changes in NF-κB activity caused by radiation, Rh2, and combination treatments were measured using a electrophoretic mobility shift assay (EMSA). The NF-κB DNA binding activity was increased in CT26/*luc* cells after 4 Gy irradiation, but suppressed by 75 μM Rh2, as shown in Figure 4.

### 2.5. Rh2 Suppresses the Expressions of NF-κB Related Proteins in CT26/luc Cells Assayed by Western Blotting

**Figure 5:**
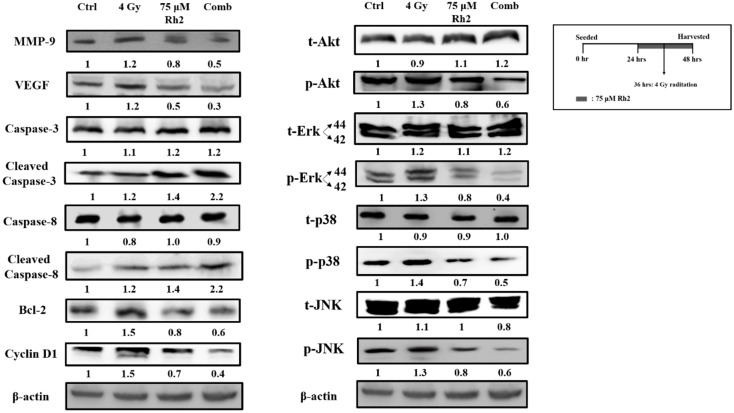
Rh2 suppresses radiation-induced proteins upstream and downstream of NF-κB and enhances the expression of caspase proteins. CT26/*luc* cells (2 × 10^6^) were seeded into 10-cm dishes for 24 h and randomly divided into four groups. The results obtained from Western blotting showed that Rh2 could reduce the expressions of NF-κB related proteins, including MMP-9, VEGF, Cyclin D1, and Bcl-2, in CT26/*luc* cells as shown in Rh2-alone and combination groups. Phosphorated ERK, AKT, JNK, and p38 of the MAPK family were all suppressed in RH2-alone and combination groups. In addition, cleaved caspase-3 and cleaved caspase-8 were both significantly increased in the combination group.

NF-κB downstream effector proteins are involved in cell proliferation, anti-apoptosis, angiogenesis, and metastasis. MAPK family proteins, including ERK, AKT, JNK, and p38, are the upstream signaling molecules of NF-κB. However, whether Rh2 could regulate the expression of these proteins is ambiguous. Figure 5 shows that expressions of phosphorylated ERK, AKT, JNK, and p38 were elevated after cells were treated with radiation but suppressed by 75 μM Rh2 and combination treatments. Expressions of NF-κB downstream effector proteins, such as MMP-9, VEGF, Bcl-2, and Cyclin D1, induced by radiation could be suppressed by Rh2. Moreover, the apoptotic-related proteins, such as cleaved caspase-3 and cleaved caspase-8, were significantly increased by combination treatment.

### 2.6. Pretreatment with Rh2 Combined with Radiation Enhances Therapeutic Efficacy in CT26/luc Tumor-Bearing Mice

**Figure 6:**
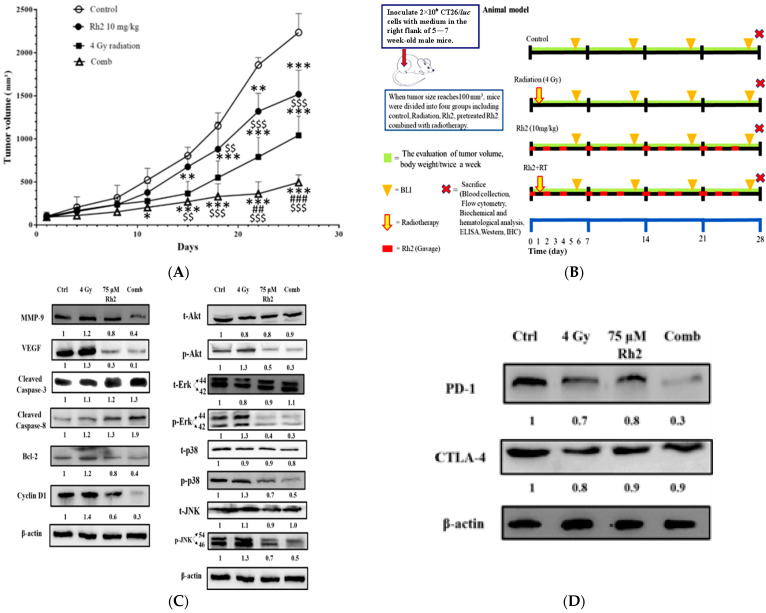
Rh2 enhances the therapeutic efficacy of radiation in CT26/*luc* tumor-bearing mice. (**A**) Flow chart of in vivo experiments. CT26/*luc* cells were subcutaneously implanted into the right flanks of the male mice. Mice were randomly divided into four groups (n = 6 per group): control, 4 Gy irradiation, Rh2 alone (10 mg/kg, gavage), and a combination of both (pretreated with 10 mg/kg Rh2 one day prior to 4 Gy irradiation). (**B**) Tumor volume was significantly inhibited in all treatment groups, especially in the combination group. (**C**) The expressions of tumor progression-related proteins, such as MMP-9, VEGF, Bcl-2, and Cyclin D1, were all increased in the radiation-alone group but were decreased in Rh2-alone and combination groups. The cleaved caspase-3 and -8 were increased in all treatment groups. Phosphorylated MAPK proteins, such as Akt, Erk, JNK, and p38, were all increased in the radiation-alone group but decreased in Rh2-alone and combination groups. (**D**) The splenocytes of the spleens were used to assay the expressions of CTLA-4 and PD-1. Both CTLA-4 and PD-1 could be slightly reduced by radiation and Rh2-alone treatments. Notably, PD-1 but not CTLA-4 was significantly reduced by the combination treatment. * *p* < 0.05, ** *p* < 0.01, and *** *p* < 0.001 for the treatment groups compared with that of the control. ^##^ *p* <0.01 and ^###^ *p*< 0.001 for Rh2-alone and combination groups compared with that of the radiation-alone group. ^$$^ *p* < 0.01 and ^$$$^ *p* < 0.001 for radiation-alone and combination groups compared with that of the Rh2-alone group.

The experimental protocol for the in vivo study is shown in Figure 6A. Since Rh2 demonstrated the radiosensitization effect in an in vitro study, we further examined the therapeutic efficacy in a CT26/*luc* tumor-bearing animal model. Tumor size was measured using a digital caliper twice a week. The tumor growth curves are shown in Figure 6B. On day 26 post-treatment, the average tumor volumes of the control, radiation-alone, Rh2-alone, and combination groups were 2200 mm^3^, 1000 mm^3^, 1500 mm^3^, and 490 mm^3^, respectively. The most significant tumor growth inhibition was found in the combination group. Body weights were monitored to evaluate the general toxicities caused by these treatments, as shown in supplemental Figure S2. The mean tumor growth delay time and combination enhancement ratios in CT26/*luc* tumor-bearing mice are shown in Table 2. The combination enhancement ratios were 2.09- and 1.56-fold for Rh2-alone and radiation-alone groups, respectively. The combination index was calculated as 0.71, as shown in Table 3. A synergistic effect was found for the combination of Rh2 and radiation, and therapeutic efficacy was enhanced. Furthermore, mice were sacrificed on day 26, and tumors were removed for ex vivo Western blotting. The expressions of MMP-9, VEGF, Bcl-2, and Cyclin D1, involved in tumor progression, were found to increase in the radiation-alone group, but they decreased in the Rh2-alone and combination groups (Figure 6C). The MAPK proteins in the NF-κB signaling pathway, such as p-Akt, p-Erk, p38, and p-JNK, were all decreased. In contrast, cleaved caspase-3 and -8, the apoptotic-related proteins, significantly increased in the combination group. These results were similar to those of the in vitro study, as shown in Figure 5. In addition, the splenocytes harvested from tumor-bearing mice were used to assay CTLA-4 and PD-1 expressions. Notably, PD-1 but not CTLA-4 was significantly reduced in the combination group, as shown in Figure 6D.

### 2.7. Rh2 Enhances Therapeutic Efficacy in a Tumor-Bearing Mouse Model via Modulating Expressions of Interleukin-12, Interleukin-18, and Interferon-γ

**Figure 7:**
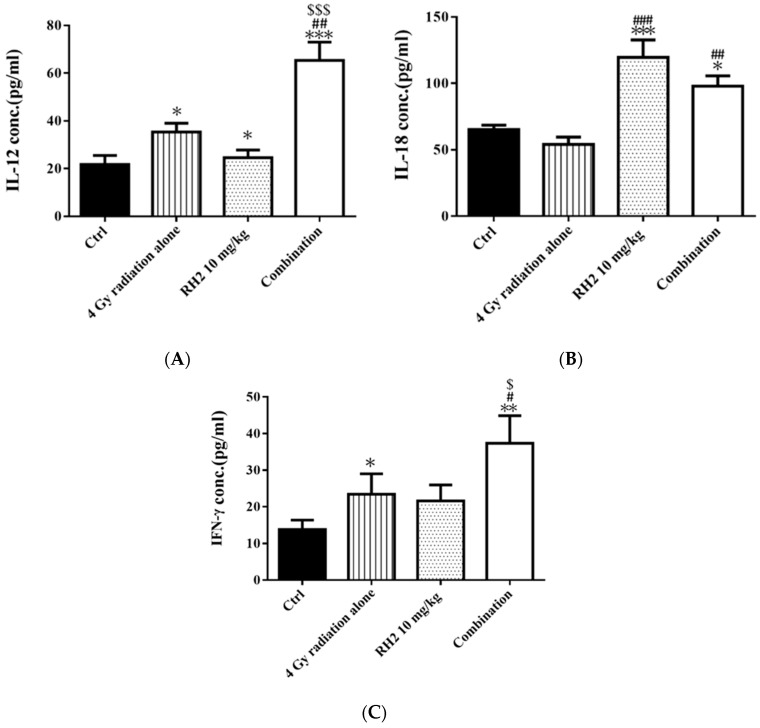
The immune responses in CT26/*luc* tumor-bearing mice after radiation-alone, Rh2-alone, and combination treatments. Mice were sacrificed on day 26, and the sera were isolated for ELISA. (**A**) IL-12, (**B**) IL-18, and (**C**) IFN-γ were all increased in Rh2-alone and combination groups. * *p* < 0.05, ** *p* < 0.01, and *** *p* < 0.001 for the experimental groups compared with that of the control. ^#^ *p* < 0.05, ^##^ *p* < 0.01, and ^###^ *p*< 0.001 for Rh2-alone and combination groups compared with that of the radiation-alone group. ^$^ *p* < 0.05 and ^$$$^ *p* < 0.001 for radiation-alone and combination groups compared with that of the Rh2-alone group.

On day 26, the blood of mice was collected via cardiac puncture, and sera were used to determine the concentrations of interleukin-12 (IL-12), interleukin-18 (IL-18), and interferon-γ (INF-γ) by enzyme-linked immunosorbent assay (ELISA). Figure 7 shows that both IL-12 and IFN-γ were significantly increased in the radiation-alone and Rh2-alone groups and were most significantly increased in the combination group. The levels of IL-18 were increased in both the Rh2-alone and combination groups.

### 2.8. Spleen Index and Lymphocyte Subset Determination

**Figure 8:**
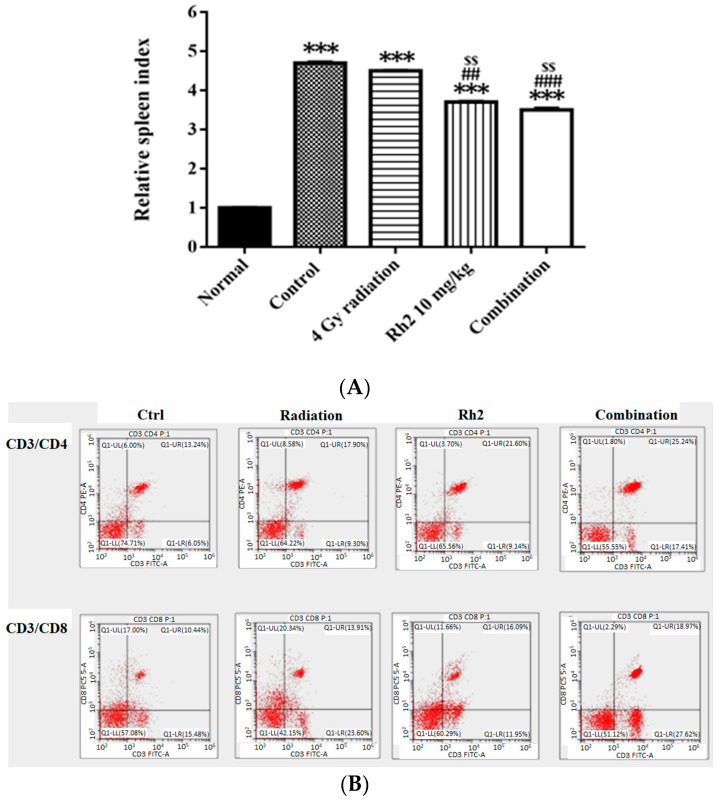
The relative spleen indices (RSIs), CD4+, and CD8+ in CT26/*luc* tumor-bearing mice after radiation-alone, Rh2-alone, and combination treatments were assayed on day 26. (**A**) The RSI in Rh2-alone and combination groups show the most significantly decreased levels compared with that of the control. (**B**) The splenocytes were used for CD4+ and CD8+ T-lymphocyte assays. The distribution ratios of the upper right regions are calculated in Table 4. *** *p* < 0.001 for the control and experimental groups compared with that of the normal. ^##^ *p* < 0.01 and ^###^ *p* < 0.001 for the experimental group was compared with the control group. ^$$^ *p* < 0.01 for Rh2 alone and combination groups compared with that of the radiation-alone group. (n = 3 per group, and the experiments were repeated three times).

On day 26, mice were sacrificed, and spleens were removed and weighed. The relative spleen index was obtained by dividing the spleen weight by the body weight of the same mouse and normalized to 1 for the normal mice. The control and treatment groups were compared with those of the normal mice (Figure 8A). The results showed that the spleen indices of all groups were higher than those of the normal mice. Notably, the spleen indices in Rh2 alone and combination groups were both significantly decreased compared with those of the control and radiation alone groups. The lymphocyte subsets were determined by flow cytometry. The results showed that the frequencies of CD4^+^ and CD8^+^ T lymphocytes were increased in mice treated with Rh2 alone and combination groups, as shown in Figure 8B and Table 4. The control group had the lowest levels of CD4^+^ and CD8^+^ T lymphocytes (13.6 ± 0.8% and 11.5 ± 1.0%). Notably, the combination group had the highest levels of CD4^+^ and CD8^+^ T cells (24.8 ± 0.5% and 20.5 ± 2.5%).

### 2.9. Immunohistochemical Staining for Proteins in Tumors and Spleens

**Figure 9:**
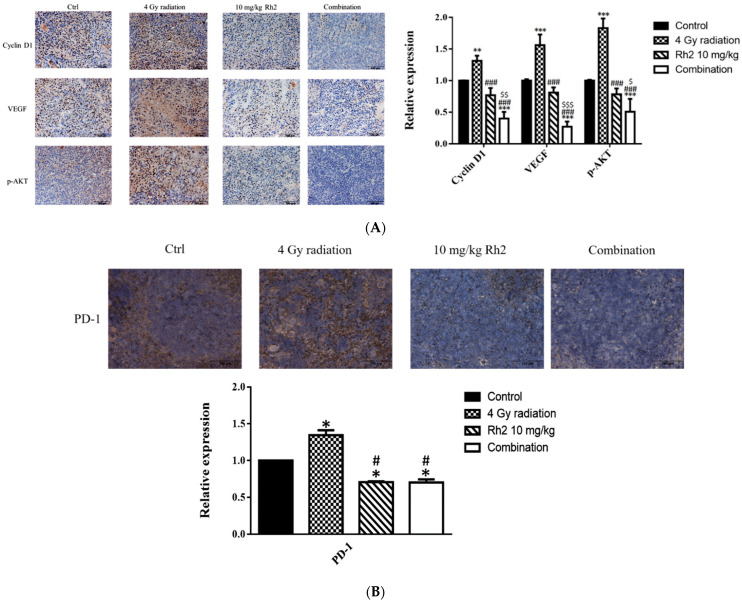
Immunohistochemical staining of proteins in tumors and PD-1 in spleens of mice sacrificed on day 26 post-treatment. (**A**) The expressions of Cyclin D1, VEGF, and p-AKT in tumors were increased by radiation alone but decreased by Rh2-alone and combination groups. Blue represents the nucleus, and brown represents the protein expression. Bars indicate 100 µm. (**B**) Expressions of PD-1 in Rh2-alone and combination groups were significantly decreased compared with those of the control and radiation-alone groups. * *p* < 0.05, ** *p* < 0.01, *** *p* < 0.001 for treatment groups compared with those of the control. ^#^ *p* < 0.05, ^###^ *p* < 0.001 for Rh2 alone and combination groups compared with the radiation-alone group. ^$^ *p* < 0.05, ^$$^ *p* < 0.01, ^$$$^ *p* < 0.001 for combination group compared with the Rh2-alone group.

The expressions of proteins associated with tumor progression in the tumor and immune checkpoint-related proteins in the spleen were assayed with immunohistochemical (IHC) staining. As shown in Figure 9A, the expressions of Cyclin D1, VEGF, and p-AKT were significantly reduced in the combination group compared with those of the control, 4 Gy-alone, and Rh2-alone groups. These results were consistent with those shown by ex vivo Western blotting. In addition, the expressions of PD-1 in spleens of both Rh2-alone and combination groups were decreased when compared with those of the control and radiation-alone groups (Figure 9B).

### 2.10. Rh2 Shows No Toxicity in Mice by Biochemical Analysis and Histopathology

In addition to monitoring body weight for general toxicity, we conducted biochemical analysis and histopathology to assess the potential toxicities of the treatments in vivo. We analyzed enzymes and proteins associated with liver functions, such as alkaline phosphatase (ALP), albumin (ALB), alanine aminotransferase (ALT), and aspartate aminotransferase (AST). Additionally, we used blood urea nitrogen (BUN) and creatinine (CRE) levels as indicators of kidney function.

**Figure 10:**
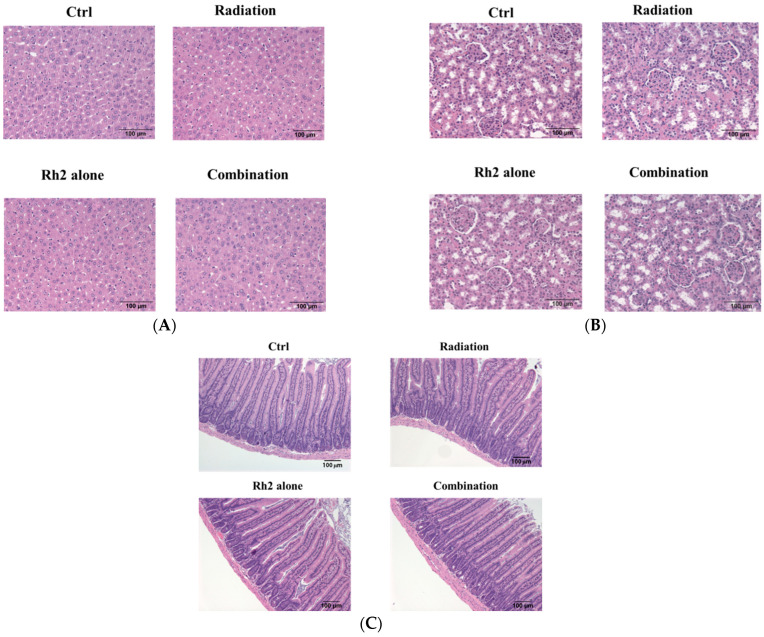
Histopathology of livers, kidneys, and small intestines of CT26/*luc* tumor-bearing mice treated with Rh2 alone, radiation alone, and combination of both. The sections were performed on day 26 post-treatment and then stained with hematoxylin and eosin. (**A**) livers; (**B**) kidneys; (**C**) small intestines. No pathological findings were observed among the control and treatment groups.

Both the liver and renal functions of mice treated with 10 mg/kg Rh2 were insignificantly different from those of the control, as shown in Table 5. The concentrations of these components were within the reference ranges, except that BUNs were slightly increased in all groups (reference ranges of biochemical data were based on the dataset provided by Charles River Laboratories, Wilmington, MA, USA). Importantly, no pathological findings were observed among the control and treatment groups (Figure 10).

## 3. Discussion

Chemotherapy to preoperative radiotherapy has been shown to have a significant benefit on local control of rectal cancer [32]. However, this combination can also cause severe side effects and reduce therapeutic efficacy due to acquired resistance. A number of plant extracts can sensitize cancer cells to radiation, in which curcumin has been found to modulate the radiation sensitivity of cancer cells by inhibiting NF-κB activity [33]. Ginseng, in which Rh2 is one of the most active components, has also shown potential in the treatment of colorectal cancer [34]. Ginseng may have a synergistic therapeutic effect when combined with ionizing radiation for the treatment of colon cancer, as shown in this study. The NF-κB signaling pathway activated by radiation, which is involved in cancer cell proliferation, anti-apoptosis, angiogenesis, invasion, and metastasis, was suppressed by ginsenoside Rh2. In addition, cytokines such as interleukins 12 and 18, interferon-γ, and CD4+ and CD8+ T-lymphocytes of the immune system may also be evoked against cancer cells. Notably, PD-1. but not CTLA-4 induced by radiation. was suppressed by Rh2 alone and Rh2 combined with radiation.

Here, Rh2 was combined with ionizing radiation to treat the CT26/*luc* murine colon carcinoma to investigate the therapeutic effects and underlying mechanisms. The cytotoxicity was analyzed using an alamarBlue assay (Figure 1A), and the radiosensitivity was analyzed by colony formation (Figure 1B). The results showed that Rh2 could synergistically enhance cell death and reduce cell survival when combined with radiation (Table 1). Furthermore, results of flow cytometry showed an increase in G0/G1 and G2/M fractions in cells treated with Rh2, while the S-phase fraction was decreased as analyzed by Figure 2. To verify the optimal sequence order for better therapeutics, CT26/*luc* cells were treated with Rh2 pre-, concurrent, and post-4 Gy radiation (Figure 3). Notably, the IC_50_ was 66 μM for cells treated with Rh2 12 h prior to radiation, which was more efficient than the others (IC_50′_s were 72 μM and 74 μM, respectively). Cells arrested at G2/M phases by Rh2 combined with radiation may be one of the mechanisms contributing to the observed synergistic effect since these are the most sensitive phases to radiation.

The NF-κB signaling pathway plays a key role in the progress of colorectal cancer (CRC) by influencing cell proliferation, apoptosis, angiogenesis, inflammation, metastasis, and drug resistance [35]. The inhibition of NF-κB has been shown to improve the effectiveness of cancer treatment with radiation [36]. Blockade of NF-κB signaling pathway, such as with sorafenib, results in suppression of downstream effectors, such as MMP-9 (invasion/metastasis), Cyclin D1 (proliferation), VEGF (angiogenesis), and Bcl-2 (anti-apoptosis), ultimately enhancing the cell killing effects [37,38]. Thus, targeted therapy against NF-κB has proven to be effective in treating colorectal cancer [39].

Here, we observed that Rh2 combined with radiation led to the most significant reduction in decreased NF-κB activity, as shown by EMSA in Figure 4. Furthermore, the downstream effectors, such as MMP-9, VEGF, Bcl-2, and Cyclin D1, were all increased by radiation alone but decreased by treatment with Rh2 alone or Rh2 combined with radiation, as shown in Figure 5. Notably, the combination group showed the lowest expressions of these four proteins compared to either treatment alone. The MAPK signaling pathways were found to be involved in the proliferation and anti-apoptosis of tumor cells post-irradiation, suggesting that MAPK signaling pathways could regulate NF-κB activity [40]. Previous research has shown that Ginsenoside can ameliorate lung inflammatory responses by inhibiting the MAPKs/NF-κB/c-Fos pathways [41]. Rh2 has also been shown to inhibit IκBα and cause NF-κB degradation in LPS-challenged mice [42]. In addition, both cleaved caspase-3 and -8 were increased by Rh2 alone and in combination treatments. These activated caspases are known to induce apoptosis in tumor cells. Furthermore, the expressions of upstream proteins involved in the activation of NF-κB in the MAPK signaling pathway were also determined with a Western blot. Phosphorylation of AKT, ERK, p38, and JNK were all increased by radiation but decreased by Rh2 alone and by Rh2 combined with radiation (Figure 5).

For the in vivo study, CT26/*luc* tumor-bearing mice were treated with 10 mg/kg Rh2 by gavage 24 h before receiving 4 Gy radiation, and Rh2 was continuously administered three times a week for 4 weeks post-irradiation. The experimental design is depicted in Figure 6A. The combination group exhibited the best tumor growth inhibition compared with Rh2-alone and radiation-alone groups. Importantly, the combination therapy showed a synergistic effect without causing general toxicity (Figure S2). Similar to the findings from the in vitro study, the levels of MMP-9, VEGF, Cyclin D1, Bcl-2, and MAPK family proteins (i.e., p-AKT, p-p38, p-ERK, and p-JNK) were all increased by radiation treatment but decreased by Rh2 alone and Rh2 combined with radiation.

Previous reports have suggested that *Panax ginseng* treatment and radiotherapy could induce immune response with immunomodulation ability [43,44]. To evaluate the immunomodulatory properties of ginsenoside Rh2 in vivo, the levels of interleukins 12 (IL-12), 18 (IL-18), and interferon-γ (IFN-γ) were evaluated with ELISA. IL-12 has been found to delay tumor growth and prolong survival in mice with bladder tumors [45]. Here, the levels of IL-12, IL-18, and IFN-γ were significantly increased in all treatment groups, except IL-18 in the radiation-alone group (Figure 7). These results suggest that both radiation and Rh2 may possess immunomodulation properties. The relative spleen indices (RSIs), an indication of immune response, of Rh2 alone and combination groups were significantly lower than those of the control and radiation-alone groups (Figure 8A). In addition, both CD4^+^ and CD8^+^ T-lymphocytes were increased significantly in the combination group compared with the control group (Figure 8B and Table 4), suggesting that the immunomodulatory function of Rh2 may play a role in therapeutic efficacy in CT26/*luc* tumor-bearing mice. Immunohistochemical staining (IHC) of tumor tissues showed that the changes in Cyclin D1, VEGF, and pAKT were similar to those in vitro results (Figure 9A), indicating that the combination of Rh2 with radiation could improve the therapeutic efficacy.

Furthermore, the expressions of PD-1 in the spleens of mice were suppressed post-treatment with Rh2 alone and combination groups (Figure 9B). No biochemical toxicity was found in the treatment groups (Table 5). Additionally, histopathological examinations of the liver, kidney, and small intestine revealed no significant difference between the control and treatment groups (Figure 10). Bioluminescent imaging (BLI) was performed once a week in the CT26/*luc* tumor-bearing mice also showed similar therapeutic outcomes (Figure S3). Overall, Rh2 enhanced radiosensitivity, suppressed the proteins involved in tumor growth, and overcame radiation-induced radioresistance. Rh2 suppressed the activity of NF-κB and its downstream effector proteins by inhibiting the radiation-induced MAPK/NF-κB signaling pathways. Cancer patients could be administered with ginseng or ginsenoside Rh2 before or during radiotherapy for synergistic therapeutic effects. Since miRNA-638 has been reported to be down-regulated to promote apoptosis and autophagy by Rh2 in several cancer cell types [18,19,20], other miRNAs may also be involved in the development of colorectal cancers, such as TGFBR1:miR-532-5P, SMAD7:miR-375, PI3KCA:miR-520a, and CD44:miR-509-3P [46]. On the other hand, long non-coding RNAs (lncRNAs) play roles in the *Kdr/Vegfα/Pten/Bdnf* interactions network in the hippocampus region and ileum tissue of the brain–gut axis [47]. LncRNAs may also participate in the development of colorectal carcinoma. The mechanisms of these miRNAs and lncRNAs involved in colorectal carcinogenesis are worth further investigation.

## 4. Materials and Methods

### 4.1. Cell Lines and Cytotoxicity of Rh2 in CT26/luc Cells

The CT26/*luc* murine colon carcinoma cell line was established as previously described [31]. Cells were maintained in RPMI-1640 medium (cat. no. SH30027.02, Hyclone, Logan, UT, USA) supplemented with 10% fetal bovine serum (cat. no. SH30080.03, Hyclone, Logan, UT, USA), 1% penicillin/streptomycin (cat. no. 15140-122, Gibco, NY, USA), and 100 µg/mL G418 (cat. no. 345810-250MGCN, Calbiochem^®^, La Jolla, CA, USA). G418 was added to maintain the stable expression of the luciferase gene. The cells were incubated at 37 °C in a humidified incubator containing 5% CO_2_.

### 4.2. Drug Preparation

20(*S*)-Ginsenoside Rh2 (Rh2, purity ≥ 98%) was purchased from ChromaDex Corp. (CAS#: 78214-33-2, Irvine, CA, USA). Rh2 was dissolved in absolute ethanol as a stock solution. The final concentration of ethanol in Rh2 was 1% when applied in the following experiments: cell cytotoxicity, electrophoretic mobility shift assay (EMSA), and Western blotting. For in vivo studies, Rh2 was suspended in phosphate-buffered saline (PBS) and administered to mice by gavage.

### 4.3. Irradiation

CT26/*luc* cells were irradiated with 4 Gy using an RS 2000 X-ray Biological Irradiator (Rad Source Technologies, Suwanee, GA, USA). The dose rate was 2.3 Gy/min. For the in vivo study, the tumor on the right hind leg of the mouse was exposed to 4 Gy, while the rest of the body was shielded with the lead grid.

### 4.4. Growth Curves of CT26 and CT26/luc Cells

A total of 5 × 10^4^ CT26 and CT26/*luc* cells per dish were seeded in 6-cm diameter culture dishes. At 0, 12, 24, 48, 72, 96, and 120 h post-culture, the cells were harvested and counted with a hemocytometer. The time points located in the exponential growth curve were used to calculate the cell growth doubling time (T_d_) using the following formula: T_d_ = (t − t_0_) × ln2/(lnN − lnN_0_), where t: the sampling time at the later exponential growth phase; t_0_: the initial sampling time of the exponential growth phase. N: the cell number of the sampling time t; and N_0_: the cell number of the initial time t_0_.

### 4.5. Bioluminescent Imaging (BLI)

BLI was performed as in our previous study and briefly described in the following [31]. For imaging in vitro, cells were diluted from 1 × 10^6^ cells/mL to 1 × 10^4^ cells/mL and seeded in the black/clear bottom 96-well plates (cat. no. 3916, Corning, NY, USA). For imaging in vivo, mice received 10 µL of a 15 mg/mL D-luciferin stock solution per gram of body weight (10 µL/g) via intraperitoneal injection for 15 min before imaging. The image acquisition time was 5 min, and Living Image Software was used to select the regions of interest (ROIs) around the tumor sites and quantify the photon numbers.

### 4.6. Cytotoxicity of Rh2 on CT26/luc Cells

A total of 1 × 10^4^ CT26/*luc* cells per well were seeded in a 96-well plate containing 100 μL RPMI 1640 medium per well. Twenty-four hours later, the medium was replaced with a fresh medium containing different concentrations of Rh2 (0, 1, 20, 50, 75, 85, 100 µM), and cultured for another 24 h. Cell viability was determined by the alamarBlue assay and detected using an ELISA reader (TECAN 200/200PRO, Durham, NC, USA). The cell viability was calculated using the equation provided by the manufacturer: (117,216 × A1 – 80,586 × A2) / (155,677 × N2 – 14,652 × N1) × 100. (A1, the absorbance of test wells at 570 nm; A2, the absorbance of test wells at 600 nm; N1, the absorbance of negative control well (medium plus alamarBlue but no cells) at 570 nm; N2, the absorbance of negative control well at 600 nm).

### 4.7. Clonogenic Formation Assay

A total of 1 × 10^6^ CT26/*luc* cells were seeded in 10-cm diameter dishes and incubated for 24 h. The cells were irradiated with 0–8 Gy alone or combined with 75 μM Rh2. Cells were trypsinized after irradiation and seeded in 6-cm dishes for 2 weeks. The colonies were fixed with methanol: glacial acetic acid (3:1) fixative and stained with 0.25% crystal violet. The surviving fraction (SF) was calculated using the following formula: SF = number of colonies counted/(number of cells plated × plating efficiency). The evaluation of the radiosensitization effect of Rh2 on CT26/*luc* cells was similar to our previous study [31].

### 4.8. Cell Cycle Analysis by Flow Cytometry

For in vitro studies, 2 × 10^6^ CT26/*luc* cells were seeded in 10-cm dishes for 24 h. The cells were treated with 75 μM Rh2 alone, 4 Gy radiation alone, and 75 μM Rh2 combined with 4Gy radiation. After treatments, cells were harvested and fixed with ice-cold 70% ethanol in PBS at −20 °C overnight. After washing with PBS twice, the cells were stained using 1 mL propidium iodide (PI) solution [20 µg/mL propidium iodide (cat. no. P4170, Sigma-Aldrich, St. Louis, MO, USA) plus 200 µg/mL RNaseA (cat. no. E866, Amresco, Dublin, Ireland) and 0.1% (*v*/*v*) triton x-100 (cat. no. 9002-93-1, Sigma-Aldrich, St. Louis, MO, USA) in PBS] in the dark for 30 min in the 37 °C incubator, and analyzed by FACScan flow cytometer (Beckman Coulter CytoFLEX, Brea, CA, USA). For in vivo studies, mice were sacrificed, and spleens were removed and ground; the splenocytes were resuspended in PBS, and centrifuged at 1200 rpm for 10 min. After removing the supernatant, 3 mL of red blood cell lysis buffer (cat. no. 20110, STEMCELL Technologies, Vancouver, BC, Canada) was added and incubated with the splenocytes for 1 min to lyse the erythrocytes. The suspension was centrifuged at 1200 rpm for 10 min, and the remaining cells were washed twice with PBS and resuspended in 30 mL easy separation buffer (cat. no. 20144, STEMCELL Technologies, Vancouver, BC, Canada). A total of 1 × 10^6^ lymphocytes were suspended in PBS, and CD4-PE (cat. no. 100407), CD8-PerCP (cat. no. 10073), and CD3-FITC (cat. no. 100305) (BioLegend, San Diego, CA, USA) fluorescent antibodies were added, incubated in the dark for 30 min, and analyzed with a FACScan flow cytometer (Beckman Coulter CytoFLEX, Brea, CA, USA). Data were analyzed using CytExpert software v2.3 (Beckman Coulter CytoFLEX, Brea, CA, USA).

### 4.9. Electrophoretic Mobility Shift Assay (EMSA)

Cell lysates were prepared with lysis buffer (50 mM Tris-HCl, pH 8.0, 120 mM NaCl, 0.5% NP-40). The nuclear protein was extracted following the procedures of the nuclear extraction kit provided by the manufacturer (cat. no. 2900, Millipore, Billerica, MA, USA). The following DNA sequences were synthesized for assessing the NF-κB activity. Sense: AGTTGAGGGGACTTTCCCAGGC; antisense: GCCTGGGAAAGTCCCCTCAAC. The NF-κB/DNA binding activity was evaluated using the LightShift Chemiluminescent EMSA kit (cat. no. 20148, Thermo Fisher Scientific, Waltham, MA, USA). The intensities of the bands were quantified by Image J 1.52a (National Institutes of Health, Bethesda, MD, USA).

### 4.10. Western Blot Analysis

Cells, tumors, and spleen lysates were prepared using lysis buffer (50 mM Tris-HCl, pH 8.0, 120 mM NaCl, 0.5% NP-40) and tissue protein extraction reagent (cat. no. 78510, TPER, Thermo Fisher Scientific, Waltham, MA, USA). An equal amount of lysates were separated by SDS-polyacrylamide gel electrophoreses (SDS-PAGE). The proteins were transferred to polyvinylidene difluoride membranes, and the membranes were blocked with 5% nonfat milk or 5% BSA in Tris-Tween buffer saline [TBST: 20 mM Tris, 150 mM NaCl, 0.1% Tween 20 (*w*/*v*)]. The membranes were incubated with antibodies against MMP-9 (AB19016), VEGF (ABS82) (Millipore, Bedford, MA, USA), cyclin D1 (cat. no. GTX108624), JNK (cat. no. 52361), phospho-JNK (cat. no. GTX24821), phospho-p38 MAPK (cat. no. GTX133460), p38 MAPK (cat. no. GTX110720), β-actin (cat. no. GTX109639), PD-1 (cat. no. GTX128435), CTLA-4 (cat. no. GTX32542; GeneTex, Irvine, CA, USA), phospho-AKT (cat. no. 4060), AKT (cat. no. 4691), phospho-p44/42 MAPK (ERK1/2) (cat. no. 4370), p44/p42 MAPK (ERK1/2) (cat. no. 4695), Bcl-2 (cat. no. 15071), cleaved caspase-3 (cat. no. 9664), caspase-3 (cat. no. 9662), cleaved caspase-8 (cat. no. 8592), caspase-8 (cat. no. 9746) (Cell Signaling Technology, Beverly, MA, USA) at 4 °C overnight. After washing with TBST, the membranes were incubated with horseradish peroxidase-conjugated secondary antibodies (Jackson Immunol Research Laboratories, West Grove, PA, USA) at room temperature for an hour. They were washed again with TBST, and the proteins of interest were visualized using the luminescence imaging system LAS-4000 (Fujifilm, Tokyo, Japan). The intensities of the bands were quantified by ImageJ 1.52a (National Institutes of Health, Bethesda, MD, USA), and β-actin was used as an internal control.

### 4.11. Animals

The 5~7-week-old male BALB/c mice were purchased from the National Laboratory Animal Center, Taiwan. Mice were housed (four mice per cage) under the following conditions: 20–26 °C, 40–70% humidity, and a 12-h light/dark cycle. All protocols followed the guidelines of the Animal Care and Use Committee at National Yang Ming Chiao Tung University with permission number IACUC1080604.

### 4.12. Therapeutic Efficacy of Rh2 Combined with Radiotherapy in the Tumor-Bearing Animal Model

A total of 2 × 10^6^ CT26/*luc* cells mixed with serum-free RPMI-1640 medium were implanted into the right flank of the mouse. When the tumor size reached about 100 mm^3^, mice were randomly divided into five groups (n = 6 per group): normal (mice without tumors and no treatment), control (mice with tumors but no treatment), radiation (4 Gy) alone, Rh2 (10 mg/kg) alone, and pretreated Rh2 combined with radiation. Tumor volume was measured by a digital caliper twice a week, and calculated with the formula: (4/3)π × (length/2) × (width/2) × (height/2) = 0.523 × (length × width × height). Control mice were administered with PBS by gavage. The experiment was performed up to 26 days post-treatment. Mice in the radiation group were irradiated with 4 Gy using RS 2000 X-ray Biological Irradiator. For the Rh2-alone group, mice received Rh2 (10 mg/kg) by gavage three times a week for 4 weeks. For the combination group, mice received Rh2 one day before 4 Gy and were continuously administered with Rh2 three times a week for up to 4 weeks.

### 4.13. Determination of Interleukin-12, Interleukin-18, and Interferon-γ

A total of 300 μL blood was collected with the cardiac puncture method from each mouse, left on ice for 1 h, and then centrifuged at 2000 rpm at 4 °C for 25 min. The serum was stored at −20 °C. The levels of interleukin-12, -18, and interferon-γ were determined using a mouse IL-12 p70 ELISA kit (cat. no. D1200, R&D System, USA), mouse IL-18 ELISA kit (cat. no. 7625, MBL, Nagoya, Japan) and mouse INF-γ ELISA kit (cat. no. MIF00, R&D System, USA). For interleukin-12 and interferon-γ, 50 μL of assay diluent was added to each well, 50 μL of the standard was added, and then incubated for two hours at room temperature. Plates were washed five times with 400 μL of wash buffer and 100 μL of conjugate reagent was added for two hours at room temperature. The washing step was repeated as before, and 100 μL of substrate solution was added for 30 min at room temperature in the dark. Finally, 100 μL stop solution was added to terminate the reaction. For interleukin-18, 100 μL of standard and sample were added to mouse IL-18 antibody-coated microwells and incubated for one hour. The well contents were aspirated, washed four times with washing solution, then 100 μL of conjugate solution was added to each well, and incubated for one hour at room temperature. The washing step was repeated as before, and then 100 μL of substrate solution was added for 30 min at room temperature. Finally, 100 μL stop solution was added. Absorbance at a wavelength of 450 nm was detected by an ELISA reader (TECAN 200/200PRO, Durham, NC, USA).

### 4.14. Spleen Index

On day 26 post-treatment, the spleens of mice were excised and weighed. The spleen index was calculated from the spleen weight divided by the body weight of each mouse. The relative spleen indices of the mice were also assayed and compared with that of the normal mouse, which was normalized as 1.

### 4.15. Biochemical Analysis

On day 26 post-treatment, mice were sacrificed, and the blood was collected via cardiac puncture. The serum was collected and analyzed for levels of alkaline phosphatase (ALP), albumin (ALB), alanine aminotransferase (ALT), aspartate aminotransferase (AST), blood urea nitrogen (BUN), and creatinine (CRE). These measurements were performed on a Fuji Dri-Chem 4000i (Tokyo, Japan). A reference range of biochemical data was obtained from the Charles River Laboratories (Wilmington, MA, USA).

### 4.16. Tissue Preparation for Histopathology

At the end of the experiments, samples of the major organs, including the liver, small intestine, kidneys, and spleen, were fixed in cold 4% paraformaldehyde, embedded in paraffin, sectioned at 5 μm, and stained with 3′,3′-diaminobenzidine (DAB) substrate kit (Abcam, ab64238), and counterstained with Mayer’s hematoxylin (ScyTek Laboratories, UT, USA). All sections were scanned by an Aperio digital pathology slide scanner (Leica Biosystems, Buffalo Grove, IL, USA).

### 4.17. Immunohistochemical (IHC) Staining

The paraffin-embedded tissue sections were heated at 60 °C for 1 h, and dewaxed in xylene (Sigma-Aldrich, St. Louis, MO, USA). The tissue sections were rehydrated in graded ethanol from 95% to 75%, and finally in phosphate buffered solution with 0.05% Tween-20 (PBST). The tissue slides were heated in 10 mM citric acid buffer with 0.05% Tween-20 (pH = 6.0) at 121 °C for 3 min using a pressure cooker for antigen retrieval, and the tissue sections were incubated with peroxidase blocking reagent [RTU, EnVision^TM^ +Dual Link System-HRP (DAB+) kit, Code K4065, Dako, CA, USA] for 5 min, then blocked with goat serum for 30 min. After blocking, the tissue sections were incubated with antibodies against cyclin D1 (cat. no. GTX108624), PD-1 (cat. no. GTX128435, GeneTex, Irvine, CA, USA), 18hosphor-AKT (cat. no. 4060, Cell Signaling Technology, Beverly, MA, USA), and VEGF (ABS82, Millipore, Bedford, MA, USA) at 4 °C overnight, followed by horseradish peroxidase (HRP)-conjugated secondary antibodies. After being rinsed with PBST, the sections were developed in 3′,3′-diaminobenzidine (DAB) substrate kit (Abcam, ab64238), and counterstained with Mayer’s hematoxylin (ScyTek Laboratories, UT, USA). All sections were scanned by an Aperio digital pathology slide scanner (Leica Biosystems, Buffalo Grove, IL, USA).

### 4.18. Statistics

All data were presented as the mean ± standard error. Student’s *t*-test was used for significance analysis between the control and experimental groups. Two-way ANOVA was used to compare the experimental groups. Differences between the means were considered significant if *p* < 0.05 (* *p* < 0.05, ** *p* < 0.01, and *** *p* < 0.001 for the experimental groups compared with that of the control, ^#^ *p* < 0.05, ^##^ *p* < 0.01, and ^###^ *p* < 0.001 for Rh2-alone and combination groups compared with that of the radiation-alone group. ^$^ *p* < 0.05, ^$$^ *p* < 0.01 and ^$$$^ *p* < 0.001 for radiation-alone and combination groups compared with that of the Rh2-alone group).

## 5. Conclusions

Rh2 acts as a radiosensitizer and shows a synergistic effect when combined with ionizing radiation for the treatment of CT26 colorectal carcinoma. The combination treatment of Rh2 and radiation may have potential as an alternative for cancer treatment in clinics.

## Figures and Tables

**Table 1:** The combined effect of 75 μM Rh2 combined with various radiation doses on CT26/*luc* cells.

Radiation (Gy)	SF_R_	SF_R_ × SF_C_	SF_R+C_
2	0.74	0.37	0.36 (synergism)
4	0.26	0.13	0.06 (synergism)
6	0.03	0.015	4 × 10^−3^ (synergism)
8	3 × 10^−3^	1.5 × 10^−3^	3.5 × 10^−4^ (synergism)

SF_R_: the surviving fraction of CT26/*luc* cells treated with radiation alone; SFc: the surviving fraction of CT26/*luc* cells treated with 75 μM Rh2 alone; SF_R+C_: the surviving fraction of CT26/*luc* cells treated with radiation plus 75 μM Rh2.

**Table 2:** Combination enhancement ratio compared to Rh2-alone and radiation-alone groups in CT26/*luc* tumor-bearing mice.

Group	Number of Mice	Mean Tumor Growth Time ^a^	Mean Tumor Growth Delay Time ^b^	Mean Tumor Growth Inhibition Rate ^c^	Combination Enhancement Ratio ^d^
Control	6	9	n.a. ^e^	n.a.	n.a.
Rh2	6	12	3	1.33	2.09
Radiation	6	16	7	1.78	1.56
Combination	6	25	16	2.78	–

^a^ Mean tumor growth time: the time at which the tumor volume reaches 400 mm^3^. ^b^ Mean tumor growth delay time: the mean tumor growth time of the treated group minus that of the control. ^c^ Mean tumor growth inhibition rate: the mean tumor growth time of the treated group/the mean tumor growth time of the control. ^d^ Combination enhancement ratio: the mean tumor growth inhibition rate of the combination group/the mean tumor growth inhibition rate of the Rh2-alone or radiation-alone group. ^e^ n.a.: not available.

**Table 3:** Tumor growth inhibition rate and combination index post-treatments in CT26/*luc* tumor-bearing mice calculated according to Chow et al. [31].

Group	Number of Mice	Mean Tumor Growth Inhibition Rate ^a^ (%)	Expected Tumor Growth Inhibition Rate ^b^ (%)	Combination Index ^c^
Control	6	–	–	–
Rh2	6	33	–	–
Radiation	6	54	–	–
Combination	6	78	69	0.71

^a^ Mean tumor growth inhibition rate: [1 − (the mean tumor volume of treated group/the mean tumor volume of control on day 26)] × 100%. ^b^ Expected tumor growth inhibition rate: [(mean tumor growth inhibition rates of Rh2 plus Radiation) − the multiplication of both] × 100%. ^c^ Combination index: (1 − mean tumor growth inhibition rate of combination)/(1 − Expected growth inhibition rate).

**Table 4:** The percentages of CD4+ and CD8+ T-lymphocytes in CT26/*luc* tumor-bearing mice after various treatments.

Group	CD4^+^ T Cell (%)	CD8^+^ T Cell (%)
Normal	24.1 ± 1.2	17.1 ± 0.6
Control	13.6 ± 0.8 ***	11.5 ± 1.0 *
Radiation	18.6 ± 1.9 ^#^	13.6 ± 0.4
Rh2	21.9 ± 1.7 ^##;$^	17.8 ± 0.9 ^#^
Combination	24.8 ± 0.5 ^###;$^	20.5 ± 2.5 ^##;$^

* *p* < 0.05, *** *p* < 0.001, all other groups were compared with that of the normal group. ^#^ *p* < 0.05, ^##^ *p* < 0.01, ^###^ *p* < 0.001, all treatment groups were compared with that of the control group. ^$^ *p* < 0.05, Rh2-alone and combination groups were compared with that of the radiation-alone group.

**Table 5:** Biochemical analysis of CT26/*luc* tumor-bearing mice was performed on day 26 post-treatment. Six mice per group were assayed.

Items	Control	Radiation	Rh2 Alone	Combination	Reference Range
Liver function					
ALP (U/L)	163 ± 12	195 ± 16	190 ± 14	193 ± 23	62–209
ALB (g/dL)	2.3 ± 0.2	2.4 ± 0.3	2.1 ± 0.1	2.5 ± 0.3	2.5–4.8
ALT (U/L)	46 ± 8	54 ± 28	26 ± 5	47 ± 9	28–132
AST (U/L)	251 ± 72	187 ± 61	129 ± 6	165 ± 37	59–247
Kidney function					
BUN (mg/dL)	33 ± 2	30 ± 2	31 ± 3	30 ± 3	18–29
CRE (mg/dL)	0.5 ± 0.06	0.5 ± 0.07	0.4 ± 0.04	0.4 ± 0.05	0.2–0.8

## Data Availability

Data is contained within the article and supplementary material.

## Supplementary Materials

The following supporting information can be downloaded at: https://www.mdpi.com/article/10.3390/ph16091188/s1.
