# Supplementary material for: Synergistic Effect of Ginsenoside Rh2 Combines with Ionizing Radiation on CT26/luc Colon Carcinoma Cells and Tumor-Bearing Animal Model
Source: Pharmaceuticals (Basel). 2023 Aug 22;16(9):1188. doi: 10.3390/ph16091188 (PMC10535731; doi:10.3390/ph16091188)
Supplement: Supplementary file 1 [file pharmaceuticals-16-01188-s001.zip › pharmaceuticals-2486638-supplementary.pdf]

Supplementary Materials:

(A)

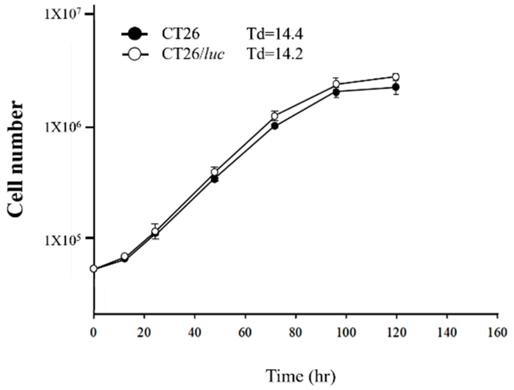

(B)

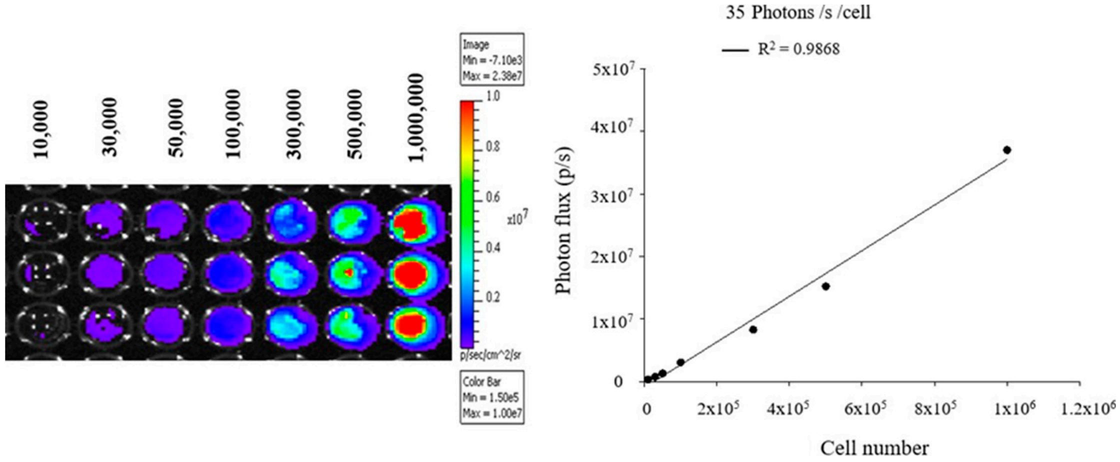

Figure S1. The cell growth curves of CT26 and CT26/*luc* cells and bioluminescence in the CT26/*luc* stable clone. (A) The cell doubling time of CT26 and CT26/*luc* cells were 14.4 and 14.2 hours, respectively, suggested no significant difference in cell growth between two cell lines. (B) After serial dilution, CT26/*luc* cells were seeded in 96-well plate and scanned with Xenogen IVIS50 optical system. The bioluminescent intensity was 35 ph/sec/cell. The correlation of the photon flux versus the cell number was R^2=0.987 by regression analysis.

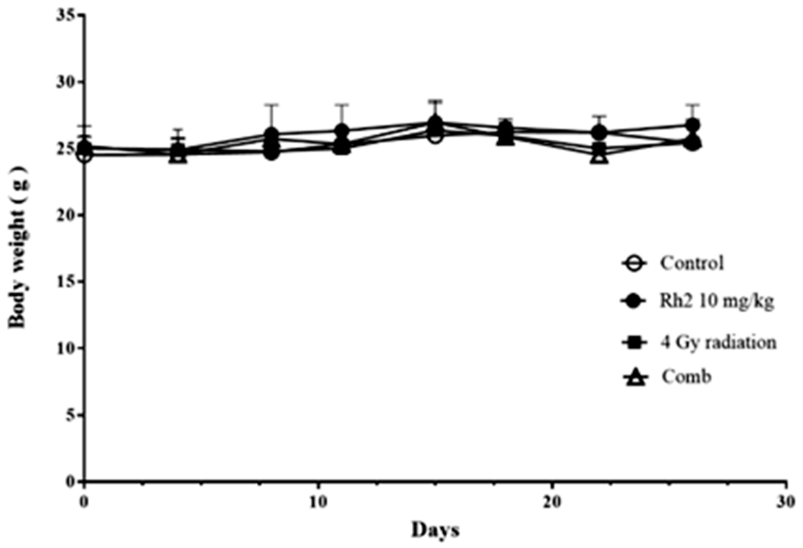

Figure S2. The body weights are monitored for all mice. The changes of body weight for all groups were within 20%, suggested no general toxicity was found in the tumor-bearing mice. (n=6 per group)

(A)

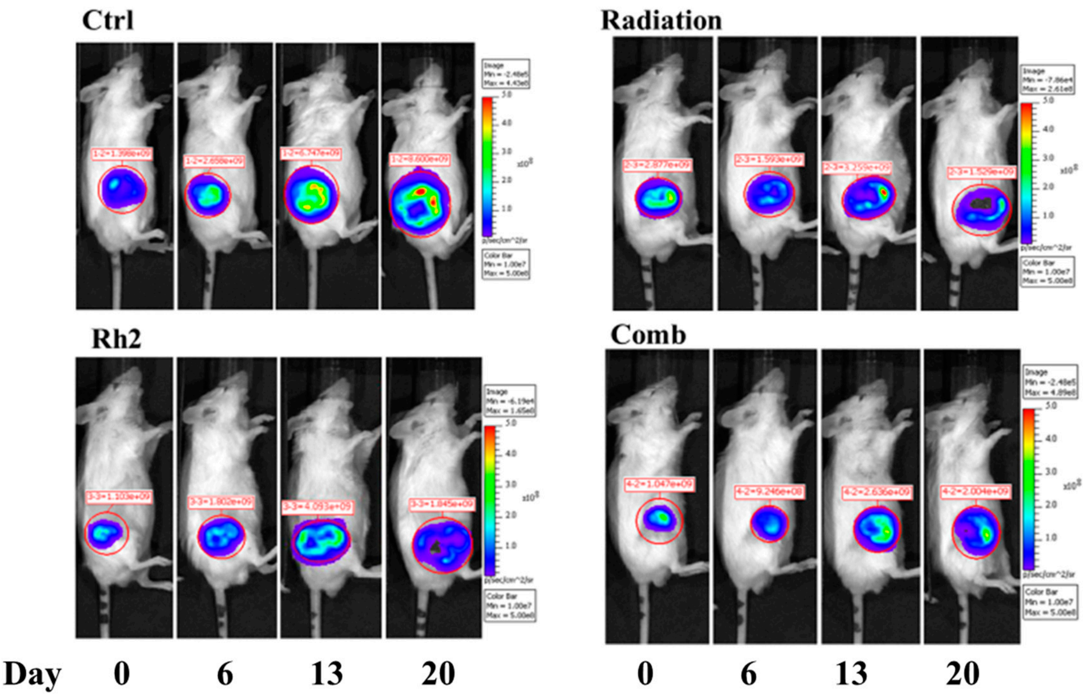

(B)

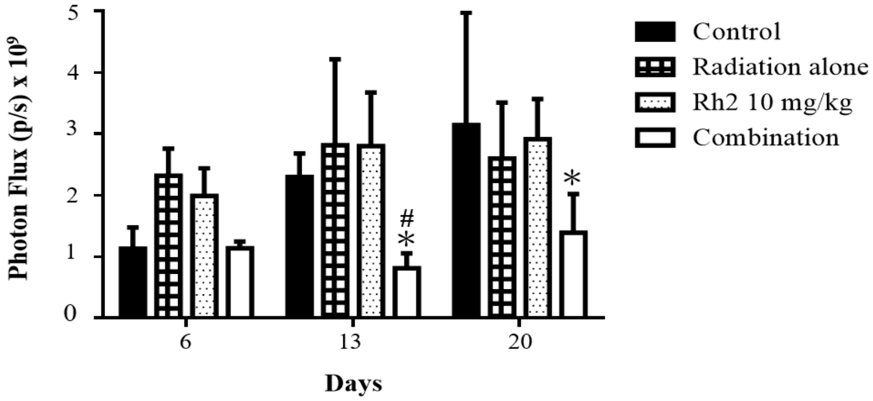

Figure S3. Therapeutic efficacy of Rh2 alone, radiation alone and combination of both was evaluated with BLI in CT26/*luc* tumor-bearing mice. (A) Tumor growth inhibition was similar to those of the tumor growth delay curves as shown in Fig. 6B. (B) Quantification of the photon fluxes from ROIs of tumor. ($^{*}p<0.05$, all treatment groups compared with the control. $^{\#}p<0.05$, Rh2 alone and combination groups compared with the radiation alone group) (n=6).

(A)

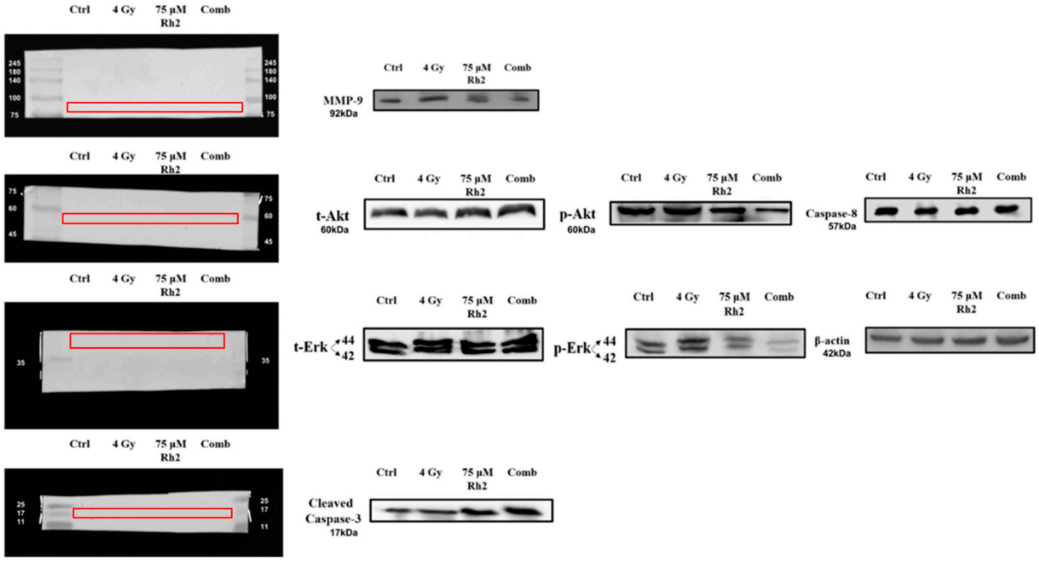
Ctrl
4 Gy
75 µM Rh2
Comb
MMP-9
92kDa
t-Akt
60kDa
p-Akt
60kDa
Caspase-8
57kDa
t-Erk
44
42
p-Erk
44
42
β-actin
42kDa
Cleaved Caspase-3
17kDa

(B)

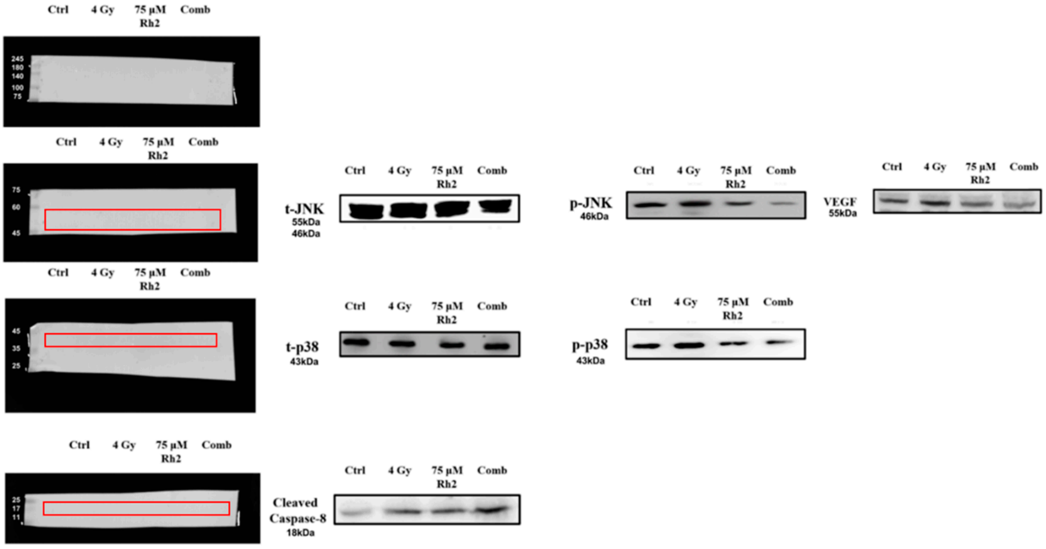
Ctrl
4 Gy
75 µM Rh2
Comb
t-JNK
55kDa
46kDa
p-JNK
46kDa
VEGF
55kDa
t-p38
43kDa
p-p38
43kDa
Cleaved Caspase-8
18kDa

(C)

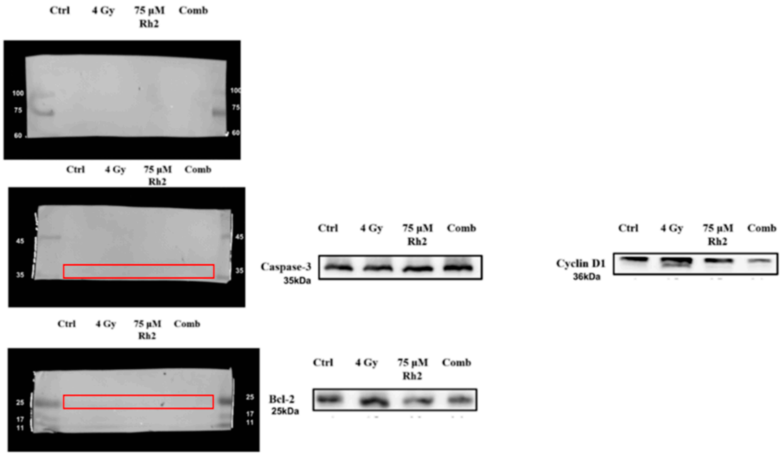

Figure S4. Original blot images presented in Figure 5. Original Western blotting membrane (light field) and blotting with different antibodies (one-to-one correlation). (A) The whole bolt images after cutting membrane at molecular weight 75 kDa for MMP-9 (92 kDa); 45 kDa for t-Akt (60 kDa), p-Akt (60 kDa), and caspase-8 (57 kDa); 25 kDa for t-Erk (44, 42 kDa), p-Erk (44, 42 kDa), and β-actin (42 kDa); the lowest left is cleaved-caspase-3 (17 kDa). (B) The whole bolt images after cutting membrane at molecular weight 45 kDa for t-JNK (55, 46 kDa), p-JNK (46 kDa), and VEGF (55 kDa); 25 kDa for t-p38 (43 kDa) and p-p38 (43 kDa); the lowest left is cleaved-caspase-8 (18 kDa). (C) The whole bolt images after cutting membrane at molecular weight 34 kDa for caspase-3 (35 kDa) and cyclin D1 (36 kDa); the lowest left is Bcl-2 (25 kDa).
